# Contrast-enhanced sonography in the diagnosis of Langerhans cell hyperplasia in children with isolated liver involvement: A case report and review of the literature

**DOI:** 10.1097/MD.0000000000050779

**Published:** 2026-09-18

**Authors:** Liu Yang, Minjuan Peng, Ni Ni, Xinyu Yan, Yuanbo Wu

**Affiliations:** a Department of Hepatobiliary Surgery, Yangxin People’s Hospital, Yangxin, China; b Department of Ultrasound, Yangxin People’s Hospital, Yangxin, China.

**Keywords:** Birbeck particles, contrast-enhanced ultrasound, Langerhans cell histiocytosis of the liver, liver

## Abstract

**Rationale:**

Isolated hepatic involvement of Langerhans cell histiocytosis (LCH) in the pediatric population is an exceedingly rare condition. Its clinical presentation is nonspecific, and its imaging features frequently overlap with those of other hepatic focal lesions, rendering preoperative diagnosis particularly challenging.

**Patient concerns:**

An 11-year-old girl presented with a chief complaint of intermittent abdominal pain.

**Diagnoses:**

Enhanced computed tomography and magnetic resonance imaging revealed multiple liver nodules. A characteristic imaging pattern was observed: during the portal venous and delayed phases, the periphery of the lesions exhibited a “wash-in and wash-out” enhancement pattern, while the central areas remained non-enhancing throughout all phases. This imaging finding provided a crucial diagnostic clue. The diagnosis of LCH was ultimately confirmed by liver biopsy, supplemented by electron microscopy and immunohistochemistry. Systemic evaluation verified isolated hepatic involvement.

**Interventions:**

The patient received immunosuppressant therapy combined with prednisone and vindesine-based combination chemotherapy.

**Outcomes:**

The patient was treated with immunosuppressant therapy and combination chemotherapy. Follow-up at 6 months indicated a favorable recovery.

**Lessons:**

This case illustrates that contrast-enhanced ultrasound can effectively delineate the unique hemodynamic characteristics of hepatic LCH lesions, serving as a valuable supplement to conventional cross-sectional imaging. In the differential diagnosis of focal liver lesions in children, particularly when there is clinical suspicion for LCH, increased recognition of these specific contrast-enhanced ultrasound features is crucial. This awareness can help prevent diagnostic delays and guide targeted pathological sampling.

## 1. Introduction

Langerhans cell histiocytosis (LCH) is a rare, nonhereditary clonal disorder of the hematopoietic system, characterized by the abnormal proliferation of CD1a+/CD207+ Langerhans cells in single or multiple organs.^[1]^ LCH occurs at all ages, most frequently in children between 1 and 4 years of age. LCH most commonly affects the bones, skin, and pituitary gland. It can also affect organs with rich reticular systems, such as the liver, spleen, and lymph nodes. LCH can be divided into single-system LCH and multisystem LCH. Isolated liver involvement is rare in LCH patients, and multisystem LCH is more common as part of the dissemination process.^[2]^ In contrast to multisystem LCH, isolated hepatic LCH is often diagnosed later due to the absence of typical manifestations such as skin rash or bone lesions. Consequently, its clinical course and prognosis remain incompletely characterized.^[3]^

The diagnosis of LCH depends on a pathological biopsy, but a needle biopsy may lead to a missed diagnosis due to sampling error. Some cases require multiple biopsies or surgical resection before diagnosis. On the other hand, the imaging findings of LCH in the liver lack specificity. Conventional ultrasound (US) often shows hepatosplenomegaly, bile duct wall thickening, or intrahepatic nodules. Computed tomography (CT) and magnetic resonance imaging (MRI) can provide more structural information, but they have limitations, such as the inability to dynamically evaluate the blood supply characteristics of the lesion.^[4]^ Contrast-enhanced ultrasound (CEUS) enables real-time visualization of tissue perfusion by intravenous injection of a microbubble contrast agent. Compared with multiphase CT and MRI, CEUS has the advantages of no ionizing radiation, convenient operation, and repeatable dynamic observation, which is especially beneficial for children who are more vulnerable to the potential harmful effects of ionizing radiation. However, the application of CEUS in children with hepatic LCH has been rarely reported.^[5]^

We report an 11-year-old girl with LCH who presented with atypical symptoms and imaging findings, including lung and liver involvement, and was subsequently diagnosed by liver biopsy. This case aims to analyze the perfusion characteristics of the lesion by CEUS and verify the diagnostic efficacy of CEUS in combination with pathological results. To our knowledge, CEUS is being used for the first time in the diagnosis of isolated hepatic LCH in children, providing new evidence for imaging assessment of this rare subtype. This study aims to suggest that CEUS can be an effective adjunctive tool for the diagnosis of hepatic LCH in children, especially in settings where multiple biopsies or radiation exposure are avoided.

## 2. Case report

In February 2022, an 11-year-old female patient was admitted to the Pediatrics Department of Yangxin County People’s Hospital of Hubei Province, China. She visited our department due to intermittent abdominal pain for more than 1 month and had no significant medical history. Physical examination revealed mild tenderness in the mid-upper abdomen. Her past medical history was unremarkable. Laboratory tests showed alanine aminotransferase 5 U/L, gamma-glutamyltransferase 164 U/L, direct bilirubin 2.4 μmol/L, indirect bilirubin 3.4 μmol/L, CRP 41.5 mg/L, and antistreptolysin O 1257 IU/mL.

**Figure 1.**
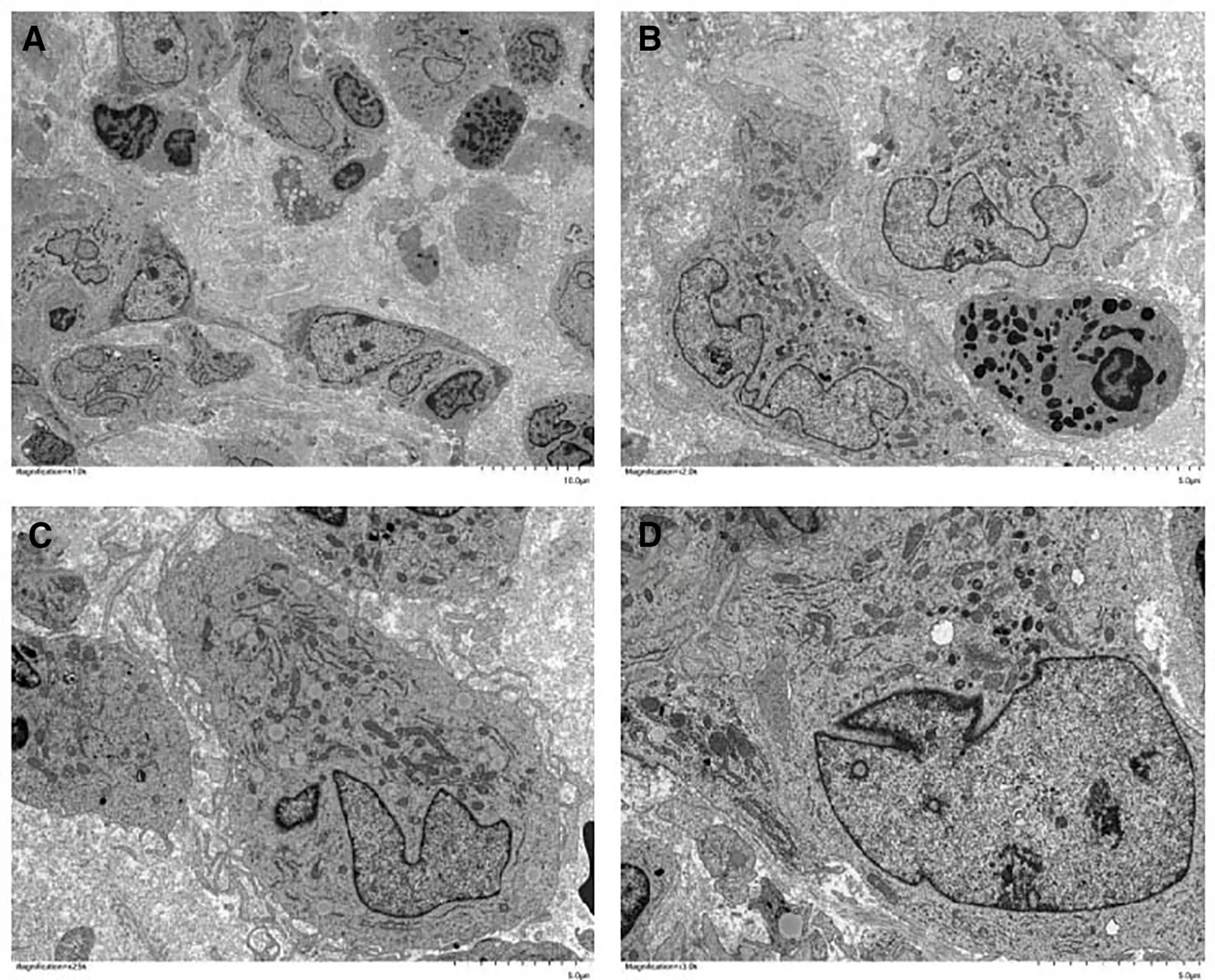
Electron microscope image. (A) Under the 10-μm scale, multiple cells with various nuclei (some of them are lobulated) and organelles in the cytoplasm can be distinguished. (B) Under the 5-μm scale, irregular nuclei and granular structures in the cytoplasm were observed. (C) Under the 4-μm scale, abundant organelles were observed in the cytoplasm, but no typical Birbeck granules were observed in the cytoplasm. (D) Under the 2-μm scale, large and irregular nuclei were observed.

Imaging examination: Contrast-enhanced CT of the upper abdomen at our hospital showed uneven density of the liver parenchyma, scattered circular hypodense shadows of different sizes, mild uneven enhancement of the lesions, and no expansion of the intrahepatic or extrahepatic bile ducts. The spleen was small. Imaging findings were suggestive of diffuse hepatic lesions, possibly of biliary origin (e.g., hamartoma). MR plain scan suggested that the signal intensity of the liver parenchyma was uneven. Multiple round long T1- and long T2-signal shadows were seen in the liver parenchyma. The largest one was located in the right lobe of the liver, measuring approximately 9 × 12 mm. The central part on T2 showed obvious high signal intensity, and the periphery showed iso- and slightly low signal intensity. The solid part showed high signal intensity on diffusion‑weighted imaging. The apparent diffusion coefficient image showed low signal intensity, and diffusion was limited. No obvious expansion of the bile ducts was observed. The gallbladder was not enlarged, and no obvious stone was seen in it. Diagnosis: multiple liver nodules with diffusion limitation (Fig. 1). CEUS: After intravenous bolus injection of SonoVue (1.2 mL), a nodule measuring approximately 13 × 11 mm was identified in the inferior segment of the right hepatic lobe. During the arterial phase (approximately 14 seconds after injection), the contrast agent reached the lesion, which demonstrated rim-like hyperenhancement at the periphery, with significantly higher enhancement intensity than the surrounding normal liver parenchyma, while the central area remained nonenhanced throughout the examination. In the late arterial phase, the peripheral enhancement began to decrease and gradually became hypoenhanced, whereas the central area remained persistently nonenhanced. During the portal venous and late phases, the peripheral enhancement further decreased with persistent hypoenhancement, and no contrast filling was observed in the central area. Overall, the lesion exhibited a characteristic enhancement pattern of “rapid peripheral enhancement followed by gradual washout, with persistent absence of central enhancement,” suggesting a neoplastic lesion or a specific infectious lesion (Video S1, Supplemental Digital Content 1, Fig. 2).

**Figure 2.**
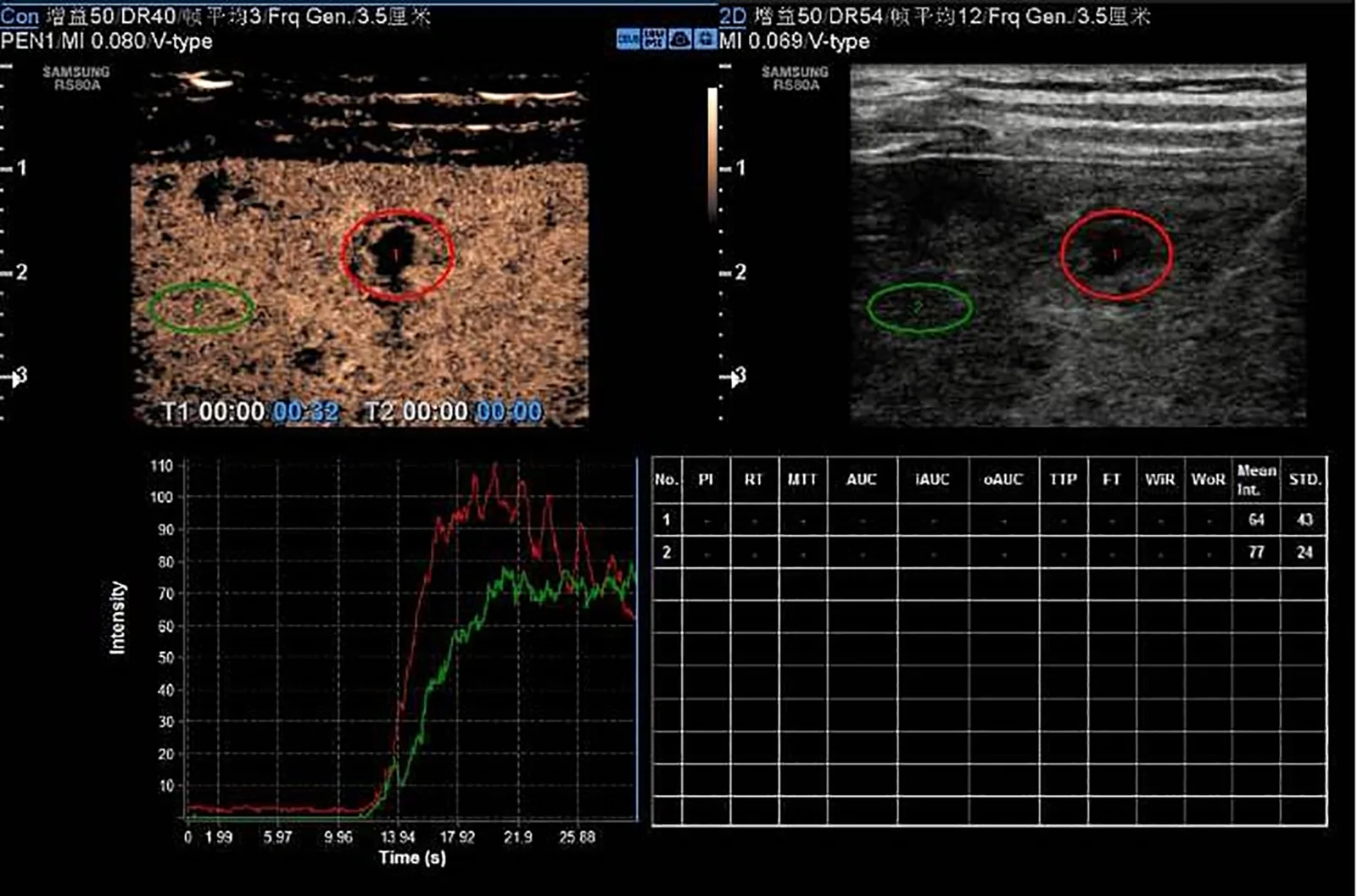
Hepatic contrast-enhanced ultrasound and quantitative analysis images. Quantitative parameters include area under the curve (AUC), inflow area under the curve (iAUC), outflow area under the curve (oAUC), time to peak (TTP), wash‑in rate (WiR), and wash‑out rate (WoR).

**Figure 3.**
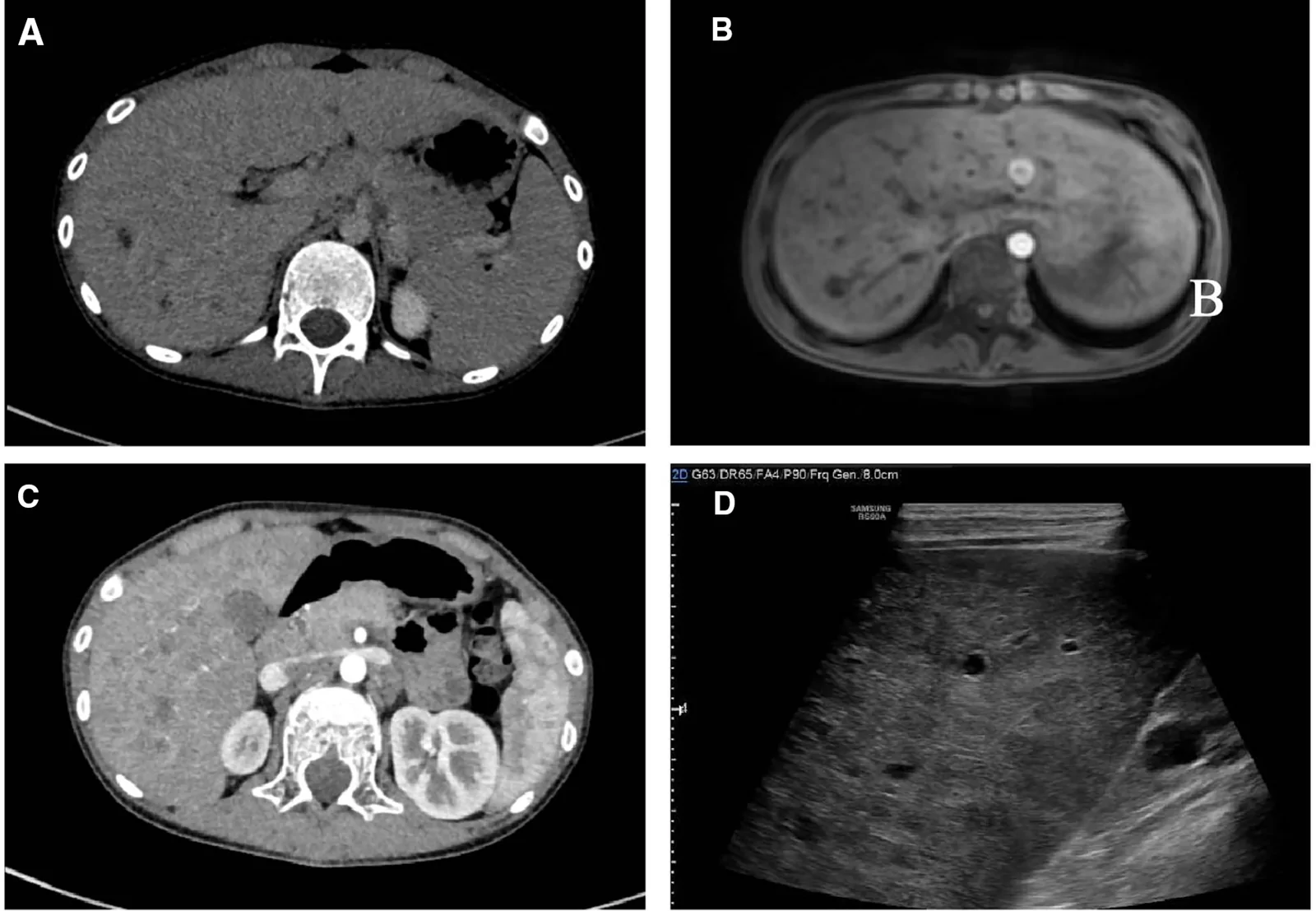
Imaging findings of liver lesions. (A) CT showed multiple circular hypodense shadows in the liver parenchyma with unclear boundaries. (B) Enhanced CT in the arterial phase showed mild enhancement. (C) Enhanced MRI showed multiple enhanced lesions in the liver parenchyma. (D) Two-dimensional ultrasound image of the liver showed multiple hypoechoic nodules with clear boundaries. CT = computed tomography, MRI = magnetic resonance imaging.

The patient was subsequently referred to a tertiary care center for further management. Enhanced MRI at the superior hospital revealed multiple nodular shadows in the liver, which may be considered inflammatory or infectious lesions, slightly thickened intrahepatic bile duct walls, and thickened lymph nodes in the hepatic portal area. Immunohistochemical analysis revealed that the lesional cells were positive for langerin, S-100, CD1a, CD45-LCA, CD68, CD163, and cyclin D1, but negative for CD21, CD23, CD35, B-Raf proto-oncogene, serine/threonine kinase (V600E), and SSTR2. The internal positive control for B-Raf proto-oncogene, serine/threonine kinase staining was positive. The Ki-67 labeling index was approximately 20%. Collectively, these findings were highly suggestive of LCH. Liver biopsy was recommended. Electron microscopy examination of the liver biopsy showed that no hepatocytes were found on ultrathin sections. It mainly contained histiocyte-like cells with large cell bodies. These cells had large cell bodies and large nuclei that were round, oval, or very irregular; a few cells were multinucleated. Nuclear euchromatin was rich, and the nucleoli were obvious. The cytoplasm was broad, with abundant mitochondria and endoplasmic reticulum, and some cells contained a few lipid droplets and lysosomes. There were many long cytoplasmic processes on the cell surface. Typical Birbeck granules were not found in the cytoplasm. Some medium-sized histiocyte-like cells were also seen. Eosinophils (mainly), neutrophils, and lymphocytes were infiltrated. Small blood vessels were dilated and filled with lymphocytes. Long spacing fibers were present between cells. Suggestion: LCH (Fig. 3).

After the diagnosis was confirmed, the patient was treated with a combination regimen of prednisone and vindesine (VDS). VDS was administered intravenously at a dose of 3 mg/m^2^ once weekly for 6 weeks, while prednisone was administered orally at 40 mg/m^2^/day for 4 weeks, followed by gradual tapering over the subsequent 2 weeks until discontinuation. At the 6-month follow-up, the patient demonstrated significant clinical improvement and remained in good condition.

## 3. Discussion

LCH is a disease that occurs primarily in children and is more common in multisystemic disorders. Isolated liver involvement in LCH is less common. Current evidence suggests that LCH may represent an immune dysregulatory disorder, potentially triggered by infections, leading to cytokine/chemokine release and, in some cases, secondary macrophage activation syndrome or hemophagocytic lymphohistiocytosis.^[2,6,7]^ If LCH is suspected clinically, histological and immunophenotypic examination of the lesion tissue should be performed.^[4,8]^

It is noteworthy that upon initial examination of this case by light microscopy, only extensive eosinophil infiltration was observed. In order to further clarify the nature of the lesion, electron microscopy was performed. Although no LCH-specific Birbeck granules were detected under electron microscopy, ultrastructural features such as cell morphology still suggested the possibility of LCH. On this basis, additional immunohistochemical tests were carried out. The results showed that the LCH-specific markers langerin (+), S-100 (+), and CD1a (+) were detected. The histopathological findings in this case were more consistent with an active cellular phase of LCH, characterized by Langerhans cell proliferation and prominent eosinophilic infiltration, without evident advanced fibrotic or sclerotic changes.^[8–10]^

Contrast-enhanced CT, MRI, and CEUS alone are insufficient for the definitive diagnosis of LCH. On contrast-enhanced CT, the liver parenchyma showed heterogeneous density, scattered round hypodense shadows of different sizes, and slightly heterogeneous enhancement of the lesions.^[11]^ On conventional MRI, multiple round long T1 and T2 signals were observed, and slight enhancement was observed at the edge of the contrast-enhanced scan.^[12]^ Contrast-enhanced CT has several advantages in the evaluation of hepatic LCH. It allows dynamic assessment of lesion enhancement, improves the detection of small hepatic lesions, facilitates evaluation of biliary and vascular involvement, and provides valuable information for biopsy planning. In addition, its short acquisition time and good patient tolerability contribute to its clinical utility. However, contrast-enhanced CT requires iodinated contrast media, which may be associated with allergic reactions and contrast-induced nephrotoxicity. Moreover, its use of ionizing radiation is a particular concern for repeated imaging during long-term follow-up in pediatric patients. In addition, CT has limited ability to characterize diffuse hepatic involvement and does not provide direct information regarding liver function.^[13]^ MRI has more prominent soft-tissue resolution, and its diagnostic accuracy is widely recognized. However, in the evaluation of pediatric LCH, MRI is limited by its relatively long acquisition time and the requirement for sustained patient cooperation. Consequently, some young children may have difficulty tolerating the examination and may require sedation or general anesthesia to complete the imaging study.

US is also an important method for determining the extent of liver involvement in LCH. US has obvious advantages, such as safety, simplicity, and rapidity, and no ionizing radiation, especially for children who need multiple follow-up visits, as it improves the convenience of repeated examinations. CEUS can supplement gray-scale and color Doppler US to improve the accuracy of lesion detection and characterization. Unlike MRI, which is susceptible to motion artifacts, the high temporal resolution of CEUS effectively mitigates this issue, allowing for real-time assessment of lesion perfusion.^[14]^ Therefore, CEUS may serve as a useful complementary contrast-enhanced imaging modality for pediatric patients, given that its microbubble contrast agents (1–3 µm in diameter) are non-nephrotoxic (applicable to patients with renal insufficiency or high-risk LCH patients) and feature minimal adverse reactions, a short intravascular half-life of only several minutes, rapid metabolism, and a high safety profile. After intravenous bolus injection of 1.2 mL of SonoVue US contrast agent, the lesion in the lower right anterior lobe of the liver was observed, with a size of about 3 × 11 mm. During the contrast-enhanced phases, the peripheral part of the lesion was enhanced in the arterial phase, but the central part was not enhanced; the peripheral part of the lesion was hypoenhanced in the portal phase and delayed phase, and the central part was not enhanced at any time. Diagnosis after examination: The CEUS pattern of the right lobe lesion was “rapid wash-in and wash-out at the edge of the lesion, and the central part was not enhanced all the time.” In this case, the enhancement pattern observed on CEUS was generally consistent with that on contrast-enhanced CT and MRI. Although none of these imaging modalities alone can establish a definitive diagnosis of hepatic LCH, their complementary use may facilitate a more comprehensive evaluation of lesion vascularity, perfusion characteristics, and structural heterogeneity. To date, few reports have focused on the use of CEUS in LCH patients with liver involvement.^[15]^ Patients with LCH with liver involvement reported in the literature generally have a poor prognosis, but our patient received first-line treatment and had a good clinical prognosis, which may be related to the mild degree of organ dysfunction at the time of initial diagnosis.

In addition, the patient was treated with a combination regimen of prednisone and VDS. This regimen is based on the standard therapeutic framework of corticosteroids combined with a vinca alkaloid.^[16]^ Prednisone suppresses disease-related inflammation primarily through its anti-inflammatory and immunomodulatory effects, whereas VDS inhibits cell proliferation by disrupting microtubule polymerization.^[17]^ Although vinblastine remains the recommended first-line vinca alkaloid for pediatric LCH, alternative vinca alkaloids, such as VDS, are occasionally used in clinical practice because of the limited availability of vinblastine in some regions of China.^[18]^ It should be emphasized that the favorable outcome observed in this case reflects the treatment response of a single patient and should not be generalized to all patients with LCH. Further studies with larger cohorts are required to validate the long-term efficacy and broader applicability of this treatment strategy.

In summary, the imaging manifestations of LCH are heterogeneous. Contrast-enhanced CT, MRI, and CEUS each provide valuable imaging information but are insufficient to establish the diagnosis of hepatic LCH independently. The present case suggests that CEUS may serve as a complementary imaging modality for the evaluation of hepatic LCH, enriching the spectrum of its imaging features and providing additional information on lesion perfusion characteristics and internal structural heterogeneity when used in conjunction with contrast-enhanced CT and MRI. In primary healthcare settings, the absence of ionizing radiation, ease of operation, and repeatability of CEUS make it a promising tool for the initial assessment and longitudinal follow-up of hepatic lesions. Furthermore, when multimodal imaging findings raise suspicion for LCH, timely referral to a tertiary care center for histopathological confirmation is essential to avoid delays in diagnosis and treatment. Further studies involving larger cohorts are warranted to clarify the clinical value of CEUS in the diagnosis and follow-up of hepatic LCH.

## Author contributions

**Investigation:** Minjuan Peng, Ni Ni, Xinyu Yan.

**Supervision:** Yuanbo Wu.

**Writing – original draft:** Liu Yang.

**Writing – review & editing:** Liu Yang.


